# Supplementary material for: Real-time scene classification of unmanned aerial vehicles remote sensing image based on Modified GhostNet
Source: PLoS One. 2023 Jun 7;18(6):e0286873. doi: 10.1371/journal.pone.0286873 (PMC10246849; doi:10.1371/journal.pone.0286873)
Supplement: Attachment — Submitted filename: Response to Reviewers.pdf [file pone.0286873.s001.pdf]

Dear Editors and Reviewers:

Thank you for your letter and for the reviewers' comments concerning our manuscript entitled "Real-time scene classification of unmanned aerial vehicles remote sensing image based on Modified GhostNet"(ID:PONE-D-23-05971). Those comments are all valuable and very helpful for revising and improving our paper as well as the important guiding significance to our researches. We have studied comments carefully and have made correction which we hope meet with approval. Revised portion are marked in yellow in the paper. The main corrections in the paper and the responds to the editors and reviewers' comments are as flowing:

Responds to the editor's comments:

1. Please ensure that your manuscript meets PLOS ONE's style requirements, including those for file naming. The PLOS ONE style templates can be found at https://journals.plos.org/plosone/s/file?id=wjVg/PLOSOne_formatting_sample_main_body.pdf and https://journals.plos.org/plosone/s/file?id=ba62/PLOSOne_formatting_sample_title_authors_affiliations.pdf

Response: We are very sorry for our careless mistakes. We have revised the manuscript as requested. We have corrected the title of the article "Real-time Scene Classification of UAV Remote Sensing Image based on Modified GhostNet" into "Real-time scene classification of unmanned aerial vehicles remote sensing image based on Modified GhostNet". In addition, we have corrected the figure citation "Figure 5" into "Fig " in the last line of the first paragraph of the Data Preprocessing section. Furthermore, some figure citations were omitted in the previous picture, and we have also made corrections in the first paragraph of Data Preprocessing. "Combination of different rotation angles and different noise is shown in Fig 3." Additional changes have also been made in Fig 7 and Fig 8 image references, such as the omission of the citation for Fig 8 (a), Fig 8 (b).

2.Please note that PLOS ONE has specific guidelines on code sharing for submissions in which author-generated code underpins the findings in the manuscript. In these cases, all author-generated code must be made available without restrictions upon publication of the work. Please review our guidelines at https://journals.plos.org/plosone/s/materials-and-software-sharing#loc-sharing-code and ensure that your code is shared in a way that follows best practice and facilitates reproducibility and reuse.

Response: Thanks for your suggestion. We have revised the problem of code sharing, and we ensure that our code is shared in a way that follows best practice and facilitates reproducibility and reuse.

3. Thank you for stating the following financial disclosure:

"Funding: This research is supported by "National Natural Science Foundation of China (NSFC)"(https://www.nsfc.gov.cn/) (Grant No. 41501370 and 62176165), and "the 5th College-enterprise Cooperation Project of Shenzhen Technology University" (https://english.sztu.edu.cn/)(Grant No. 2021010802014)."

Please state what role the funders took in the study. If the funders had no role, please state: "The funders had no role in study design, data collection and analysis, decision to publish, or preparation of the manuscript."

If this statement is not correct you must amend it as needed.

Please include this amended Role of Funder statement in your cover letter; we will change the online submission form on your behalf.

Response: Thanks for your suggestion. We have revised Role of Funder statement "The funders had no role in study design, data collection and analysis, decision to publish, or preparation of the manuscript." We have made the necessary additions to the end of the main text. Moreover, we have also added the following funding agencies and grant information:
"National Natural Science Foundation of China (NSFC)" (Grant No. 42001393）
"Shenzhen Science and Technology Innovation Program" (Grant No. JCYJ20220530152817039).

4. We note that Figure 3 in your submission contain [map/satellite] images which may be copyrighted. All PLOS content is published under the Creative Commons Attribution License (CC BY 4.0), which means that the manuscript, images, and Supporting Information files will be freely available online, and any third party is permitted to access, download, copy, distribute, and use these materials in any way, even commercially, with proper attribution. For these reasons, we cannot publish previously copyrighted maps or satellite images created using proprietary data, such as Google software (Google Maps, Street View, and Earth). For more information, see our copyright guidelines: http://journals.plos.org/plosone/s/licenses-and-copyright.

We require you to either (1) present written permission from the copyright holder to publish these figures specifically under the CC BY 4.0 license, or (2) remove the figures from your submission:

a. You may seek permission from the original copyright holder of Figure 3 to publish the content specifically under the CC BY 4.0 license.

We recommend that you contact the original copyright holder with the Content Permission Form (http://journals.plos.org/plosone/s/file?id=7c09/content-permission-form.pdf) and the following text:

"I request permission for the open-access journal PLOS ONE to publish XXX under the Creative Commons Attribution License (CCAL) CC BY 4.0 (http://creativecommons.org/licenses/by/4.0/). Please be aware that this license allows unrestricted use and distribution, even commercially, by third parties. Please reply and provide explicit written permission to publish XXX under a CC BY license and complete the attached form."

Please upload the completed Content Permission Form or other proof of granted permissions as an "Other" file with your submission.

In the figure caption of the copyrighted figure, please include the following text: “Reprinted from [ref] under a CC BY license, with permission from [name of publisher], original copyright [original copyright year].”

b. If you are unable to obtain permission from the original copyright holder to publish these figures under the CC BY 4.0 license or if the copyright holder’s requirements are incompatible with the CC BY 4.0 license, please either i) remove the figure or ii) supply a replacement figure that complies with the CC BY 4.0 license. Please check copyright information on all replacement figures and update the figure caption with source information. If applicable, please specify in the figure caption text when a figure is similar but not identical to the original image and is therefore for illustrative purposes only.

The following resources for replacing copyrighted map figures may be helpful:

USGS National Map Viewer (public domain): http://viewer.nationalmap.gov/viewer/

The Gateway to Astronaut Photography of Earth (public domain): http://eol.jsc.nasa.gov/sseop/clickmap/

Maps at the CIA (public domain): https://www.cia.gov/library/publications/the-world-factbook/index.html and https://www.cia.gov/library/publications/cia-maps-publications/index.html

NASA Earth Observatory (public domain): http://earthobservatory.nasa.gov/

Landsat: http://landsat.visibleearth.nasa.gov/

USGS EROS (Earth Resources Observatory and Science (EROS) Center) (public domain): http://eros.usgs.gov/#

Natural Earth (public domain): http://www.naturalearthdata.com/

Response: Thanks for your careful checks. We have removed the Figure 3 that contain [map/satellite] images which may be copyrighted. And we have also removed the last line in the second paragraph of the Experiment section “Some samples in the dataset are shown in Fig 3.”

5. Please review your reference list to ensure that it is complete and correct. If you have cited papers that have been retracted, please include the rationale for doing so in the manuscript text, or remove these references and replace them with relevant current references. Any changes to the reference list should be mentioned in the rebuttal letter that accompanies your revised manuscript. If you need to cite a retracted article, indicate the article’s retracted status in the References list and also include a citation and full reference for the retraction notice.

Response: Thanks for your suggestion. We have checked and revised the reference list. We have removed two references “Simonyan K, Zisserman A. Very Deep Convolutional Networks for Large-Scale Image Recognition. CoRR. 2014;abs/1409.1556.” and “He K, Zhang X, Ren S, Sun J. Deep Residual Learning for Image Recognition. 2016 IEEE Conference on Computer Vision and Pattern Recognition (CVPR). 2015; p. 770–778.”

Moreover, we have corrected the reference "Simonyan K, Zisserman A. Very Deep Convolutional Networks for Large-Scale Image Recognition. CoRR. 2015;abs/1409.1556. " into "Simonyan K, Zisserman A. Very deep convolutional networks for large-scale image recognition. arXiv preprint arXiv:14091556. 2014;.", and we have corrected the reference "Lin M, Chen Q, Yan S. Network In Network. CoRR. 2013;abs/1312.4400. " into "Lin M, Chen Q, Yan S. Network in network. arXiv preprint arXiv:13124400.2013;."

Additional Editor Comments:

The application of UAV can be enriched by description of the introduction section. The authors can refer to papers

Affum, E. A., Adigun, M. O., Boateng, K. A., Ajagbe, S. A., Addo, E. (2022). Enhancing UAV Communication Performance: Analysis Using Interference Based Geometry Stochastic Model and Successive Interference Cancellation. In: Gervasi, O., Murgante, B., Hendrix, E.M.T., Taniar, D., Apduhan, B.O. (eds) Computational Science and Its Applications – ICCSA 2022. ICCSA 2022. Lecture Notes in Computer Science, vol 13375. Springer, Cham. https://doi.org/10.1007/978-3-031-10522-7_17

Response: As suggested by the editor, we have added this reference in the third line of the introduction section as reference 3. "Unmanned Aerial Vehicles (UAVs) as a remote sensing platform are used in various applications, such as search and rescue [1], disaster evaluation , and traffic monitoring [2], wireless communications [3]."

Responds to the reviewer's comments:

1. Is the manuscript technically sound, and do the data support the conclusions?

The manuscript must describe a technically sound piece of scientific research with data that supports the conclusions. Experiments must have been conducted rigorously, with appropriate controls, replication, and sample sizes. The conclusions must be drawn appropriately based on the data presented.

Reviewer #1: Yes

Reviewer #2: Yes

Response: Thank you for taking the time to review our manuscript and for your positive feedback. We are pleased to hear that you found our work technically sound and that the data supported the conclusions.

2. Has the statistical analysis been performed appropriately and rigorously?

Reviewer #1: Yes

Reviewer #2: Yes

Response: Thank you for your positive feedback. We are glad to hear that the manuscript and the statistical analysis have been found to be technically sound and rigorous by both reviewers.

3. Have the authors made all data underlying the findings in their manuscript fully available?

The PLOS Data policy requires authors to make all data underlying the findings described in their manuscript fully available without restriction, with rare exception (please refer to the Data Availability Statement in the manuscript PDF file). The data should be provided as part of the manuscript or its supporting information, or deposited to a public repository. For example, in addition to summary statistics, the data points behind means, medians and variance measures should be available. If there are restrictions on publicly sharing data—e.g. participant privacy or use of data from a third party—those must be specified.

Reviewer #1: No

Response: Thank you for your suggestion. We have made all data underlying the findings in our manuscript fully available without restriction, as required by the PLOS Data policy. And we have made the necessary additions to the end of the main text: " The data used in this study are available from the NWPU-RESISC database (https://hyper.ai/datasets/5449), UCMerced database (https://hyper.ai/datasets/5431), and AID database.(https://hyper.ai/datasets/5446). "

Reviewer #2: Yes

Response: Thank you for your suggestion.

4. Is the manuscript presented in an intelligible fashion and written in standard English?

PLOS ONE does not copyedit accepted manuscripts, so the language in submitted articles must be clear, correct, and unambiguous. Any typographical or grammatical errors should be corrected at revision, so please note any specific errors here.

Reviewer #1: Yes

Reviewer #2: Yes

Response: Thank you for your positive feedback on the clarity and standard of English in our manuscript. We will ensure that any remaining typographical or grammatical errors are corrected during the revision process.

5. Review Comments to the Author

Please use the space provided to explain your answers to the questions above. You may also include additional comments for the author, including concerns about dual publication, research ethics, or publication ethics. (Please upload your review as an attachment if it exceeds 20,000 characters)

Reviewer #1: The authors should write UAV in full for the paper title.

Response: Thank you for your valuable suggestion. We have revised the title accordingly to reflect this change. "Real-time scene classification of unmanned aerial vehicles remote sensing image based on Modified GhostNet".

Under the Abstract section, what does the authors mean by M as the measurement unit? Is it MB or Mb or M what?
Response: The "M" appearing earlier refers to MFLOPs, while the "M" appearing later refers to MB. We have revised the abstract “Compared with the basic GhostNet, the Floating Point Operations (FLOPs) are reduced from 7.85MFLOPs to 2.58MFLOPs, the memory is reduced from 16.40MB to 5.70MB, and the inference speed is improved by 18.86%.” MFLOPs stands for "Mega Floating Point Operations per Second". It is a unit of measurement used to express the computational complexity of a mathematical operation or algorithm, particularly in the context of computer vision and machine learning. One MFLOP is equivalent to performing one million floating-point operations per second.

Generally,
* Placing the Figures very far from where it was cited in the body of the paper makes it difficult to really see if the Figures are in order for the purpose mentioned or not. E.g. There are 2 Figures (a & b) on the page referred to for Figure 1.
Response: We sincerely thank the editor for the careful reading. It is important to ensure that the figures are placed close to their corresponding citations in the paper for better readability and understanding. We have repositioned the Fig 6 to be closer to the corresponding citation in the paper.

* Table 1 content are values for different parameters and not a model.
Response: It is really true as the reviewer suggested that there is confusion in Tabel 1. We have made the necessary revisions to clarify the content of Table 1. “The overall architecture is shown in Table 1.” We have corrected the table name “Table 1. Structure of original GhostNet model.” into “Table 1. Original overall architecture of GhostNet.” And we have corrected the table name “Table 2. Structure of Modified GhostNet model.” into “Table 2. Modified overall architecture of GhostNet.”

* From the 2 bottlenecks shown in Figure 2, there seems to be no difference between the 2 bottlenecks, apart from the number of iterations (2 & 3 respectively). If that is the case, then the authors can just use one figure and include in the explanation the 2 & 3 iterations.
Response: We have revised Figure 2 to clarify the differences between the two bottlenecks. One bottleneck consists of two Ghost modules, while the other bottleneck includes a depthwise convolution with a stride of 2 in the middle of the two Ghost modules. We have also added corresponding content in the third line of paragraph 3 of the GhostNet Model section to better explain the differences between the two bottlenecks. “Ghost bottleneck is mainly composed of two Ghost modules stacked. For stride of 2, the shortcut path uses the downsampling layer and inserts depthwise convolution with the stride of 2 in the middle of the Ghost module.”

* First paragraph under Discussion section, the authors should include correct measurement units for their respective evaluation metrics.

Response: We are very sorry for our negligence of the measurement units. In the first paragraph under the Discussion section, we have added the correct measurement units for each evaluation metric used in the paper. "The calculation amount was 2.58MFLOPs, the memory usage was 5.7MB, the predicted time on Jetson TX2 was 42.6ms, the Acc On the UCMerced dataset was 96.19, and the Acc on the AID dataset was 92.05."

* Correct the 1st phrase in your conclusion section. "Based on the original GhostNet,..."

Response: Thanks for your careful checks. We are sorry for our careless. We have made the modification in the conclusion section: "Based on the original GhostNet,..."

* The authors should be consistent about their evaluation metrics. Under Discussion section, it was stated that "The evaluation metrics examined in this study were FLOPs, memory usage, predicted time and Acc...".

However, under Conclusion and Abstract section, it was stated that "The performance of the Modified GhostNet is evaluated in terms of calculation amount, memory usage, and classification speed."

Response: Thank you for pointing out the inconsistency. We have revised the Abstract, Introduction, Discussion, and Conclusion sections to use the same evaluation metrics. FLOPs (Floating Point Operations Per Second) is a measure of the computational complexity of a neural network model. It measures the number of floating-point operations that are required to perform a forward pass through the model. Memory usage refers to the amount of memory required to store the model's parameters and intermediate data during the training and inference process. Predicted time or inference time is the time required to make a prediction on a single input example using the trained model. Acc, or accuracy, is a metric that measures the proportion of correctly classified examples over the total number of examples. Calculation amount is similar to FLOPs and refers to the total number of arithmetic operations required to perform a forward pass through the model. Classification speed is similar to predicted time and refers to the time required to make predictions on a batch of input examples. The modified sentences are:

Abstract: " Compared with the basic GhostNet, the Floating Point Operations (FLOPs) are reduced from 7.85MFLOPs to 2.58MFLOPs, the memory is reduced from 16.40MB to 5.70MB, and the predicted time is improved by 18.86\%. Our modified GhostNet also increases the average accuracy(Acc)(4.70\% in AID experiments, 3.39\% in UCMerced experiments)."

Introduction: "The performance of the Modified GhostNet is evaluated in terms of FLOPs, memory usage, predicted time, and Acc. "

Discussion: "The evaluation metrics examined in this study were FLOPs, memory usage, predicted time, and Acc. "

Conclusion: "The performance of the Modified GhostNet is evaluated in FLOPs, memory usage, predicted time, and Acc. "

Reviewer #2:

The MobileNet V3 (large or small) models was not used for all possible analysis- it was selective e.g. Tables 6, 7 and 8.

Response: Thanks for the reviewers' comments. It is really true as reviewer suggested that we did not use MobileNet V3 (large or small) models for all possible analysis. We have removed MobileNetV3-Large model in Table 6, and we have compared MobileNetV3-Small model as a lightweight network, which is the focus of our study.

I don't think your co-authors or the paper reviewers should be acknowledged at the end of the paper. Kindly remove.

Response: Thank you for your suggestion. We have removed the acknowledgment section from the paper before submitting the final version.

In Figure 12, the third dataset is not shown.

Response: We sincerely appreciate the valuable comments. We have made the corresponding clarification in the second line of the last paragraph in the Results section: "Weight transfer is a technique commonly used in transfer learning, where a pre-trained model on a large dataset is used as a starting point for training a model on a smaller dataset. NWPU-RESISC dataset is a larger dataset, while UCMerced and AID datasets are smaller." Therefore, we used weight transfer with NWPU-RESISC dataset as pre-training weights and fine-tuned the network model for UCMerced and AID datasets. Figure 12 did not show the performance on the NWPU-RESISC dataset because it was used only for pre-training and not for fine-tuning. Therefore, the results on the NWPU-RESISC dataset would not reflect the effectiveness of the fine-tuning technique used on the UCMerced and AID datasets.

Limit keywords to five (5).

Response: Thank you for your suggestion.We have revised the manuscript to limit the number of keywords to five.

6. PLOS authors have the option to publish the peer review history of their article (what does this mean?). If published, this will include your full peer review and any attached files.

If you choose "no", your identity will remain anonymous but your review may still be made public.

Do you want your identity to be public for this peer review? For information about this choice, including consent withdrawal, please see our Privacy Policy.

Reviewer #1: Yes: Dr Olukayode Oki

Response: We appreciate for Dr Olukayode Oki's warm work earnestly, and hope that the correction will meet with approval. Once again, thank you very much for your comments and suggestions.

Reviewer #2: No

Response: We respect your decision to not have your identity disclosed in the peer review history. We appreciate for Reviewer's warm work earnestly, and hope that the correction will meet with approval. Once again, thank you very much for your comments and suggestions.

Sincerely,
Jinzhou Cao.
Institution and address: College of Big Data and Internet,
Shenzhen Technology University, Shenzhen, China
Email: caojinzhou2011@gmail.com
